# Implementing a Canadian shared-care ADHD program in Beijing: Barriers and facilitators to consider prior to start-up

**DOI:** 10.1186/s12888-022-03955-7

**Published:** 2022-05-05

**Authors:** Sayna Bahraini, Alexander R. Maisonneuve, Yirong Liu, André Samson, Qian Ying, Fei Li, Li Yang, Philippe Robaey

**Affiliations:** 1 Children’s Hospital of Eastern Ontario, University of Ottawa, Ottawa, Canada; 2 Peking University Sixth Hospital, Peking University Institute of Mental Health, National Clinical Research Center for Mental Disorders (Peking University Sixth Hospital), NHC Key Laboratory of Mental Health (Peking University), Beijing, 100191 China; 3 Faculty of Education, University of Ottawa, Ottawa, Canada; 4 Developmental and Behavioral Pediatric Department and Child Primary Care Department, Ministry of Education, Shanghai Key Lab for Children’s Environmental Health, Xinhua Hospital, Shanghai Jiaotong University School of Medicine, Shanghai, China

**Keywords:** ADHD, Shared mental health care, Barriers and facilitators, Grounded theory, Context

## Abstract

**Background:**

The shared care pathway for ADHD is a program developed in Canada with two main strategies: (a) implement a shared care pathway between general practitioners (GPs) and specialists, and (b) step up or down care so that the patient is treated at the most appropriate level of care, depending on the complexity or outcome of their illness. The current study aims to identify the challenges and facilitators of implementing this program in a Chinese mental health service setting.

**Methods:**

Two focus groups were conducted using semi-structured interviews with a total of 7 health care providers in Beijing. An adapted grounded theory methodology using open-ended, axial and selective coding was used for data analysis.

**Results:**

We identified three main levels related to barriers and facilitators: (1) a sociocultural level of patients' and health care providers' perspectives; (2) a structural level related to internal and external organizational environments; (3) and the level of the intervention itself with its characteristics. The project is generally aligned with the mandates and goals of the health system, but two of the main obstacles are the varying qualifications of physicians in hospitals of different levels, implying different needs and flexible and adapted training programs, and the lack of appropriate patient referral systems between the different hospital levels.

**Conclusion:**

Our study highlights the importance of consultation to obtain a "lay of the land" for deciding on the implementation steps of an a priori well accepted model of care.

**Supplementary Information:**

The online version contains supplementary material available at 10.1186/s12888-022-03955-7.

## Introduction

Attention Deficit Hyperactivity Disorder (ADHD) is one of the most common chronic childhood neurodevelopmental disorders. With an estimated prevalence of 5.7% of children in China [1], the number of children with ADHD in urban areas alone is around 7.1 million. ADHD is characterized by developmentally inappropriate inattention and/or hyperactivity/impulsivity and persists into adulthood in approximately 20% of those diagnosed in childhood [2]. This disorder is often associated with academic underachievement [3], emotional lability [4], and behavioural problems [5]. A diagnosis of ADHD increases the risk of death about twofold compared to people without ADHD, primarily due to unnatural death, especially accidents [6]. ADHD that persists into adulthood can have negative consequences in an individual’s personal (difficulty with relationships and an increased risk of substance abuse) and professional life (difficulty holding a job, absenteeism) [7]. Although ADHD is chronic and often comorbid, it is also treatable. There is considerable evidence to support the efficacy and effectiveness of ADHD treatments [8]. Although evidence for pharmacological treatments is somewhat stronger than that for non-pharmacological approaches [9], ADHD guidelines [10, 11], including Chinese guidelines [12], endorse a multi-modal approach combining medication, education and behavioural therapy. Optimal outcomes in ADHD treatment depend on adherence to guidelines and coordination of different interventions that must be planned according to patient needs.

In the Chinese urban setting, there are several challenges to the implementation of good-quality ADHD care, resulting in delayed initiation and/or poor follow-up care for most patients. In these settings, primary care is provided by general practitioners (GPs) who, for the most part, do not consider themselves sufficiently competent to manage the diagnosis and treatment of children and adolescents with ADHD. One of the many possible reasons is that general practitioners receive only about 20 h of training in general clinical psychiatry at the university level. As a consequence, patients often do not trust GPs and seek out a specialist directly. Patients predominantly consult specialists in upper-level hospitals without registering with and being referred by a GP. However, there is an extreme shortage of specialists who can manage ADHD patients. We estimated that there are in China about 500 ADHD-trained physicians for about 200 million children (ratio of 1:400,000). This shortage is exacerbated by the lack of a process to refer cases that require more complex care to specialists. As a result, patients have to wait a long time for their first or follow-up visits with a specialist. In fact, the vast majority of children with ADHD do not receive the care they need. In addition, those who are diagnosed and treated receive care in higher-level hospitals concentrated in urban areas.

These challenges must be seen in the context of major changes in the health care system since the economic reforms of the 1980s. Moving away from the rural Community Medical System and urban company-owned hospitals, China uses now a three-tiered system wherein each increasing tier is dependent on the severity of a patient’s illness [13]. The first tier of care, Level 1, refers to local hospitals that use basic equipment and offer basic care. District, municipal, or provincial hospitals, referred to as Level 2 hospitals, have more sophisticated equipment when compared to the former level. Finally, ministry-owned central hospitals, or Level 3 hospitals, offer a wide range of specialized cares, having the best available equipment in China. These hospitals are comparable to university or research hospitals in North America. Just as there is a wide disparity in the level of care depending on the type of hospital, the level of education of health care providers in China also varies widely. Currently, three levels of education are available, each level dictating where a physician can practice. Those holding a primary education level (1–3 years) are able to work as “village doctors” [13]. Individuals with secondary education (2–3 years) are able to work at Level 1 and Level 2 hospitals. Physicians holding tertiary education (3–11 years) work in Level 2 or Level 3 hospitals [14]. The level of care a patient receives, including the ability to prescribe medications, is highly dependent on a physician's level of education. Some physicians still require additional training to meet the requirements of the hospital level in which they are currently employed.

To address the insufficient training for GPs and the shortage of specialists in Canada (although to a much lesser degree than in China), the Canadian Psychiatric Association and the College of Family Physicians of Canada have championed collaboration between psychiatrists and family physicians [15]. This model of shared care can be generalized to other health care professionals such as pediatricians, nurses, etc. and effectively improve access to mental health care [8]. GPs can play a crucial role in treating patients with uncomplicated ADHD, allowing specialists to treat patients with complicated ADHD instead, such as those with other developmental and psychiatric problems [16]. With this in mind, the ADHD Shared Care Program was developed in Canada based on four essential components: care pathway, shared care, stepped care and standardization. A care pathway is an evidence-based, multidisciplinary organization of care for a defined group of patients (e.g., with a diagnosis of ADHD) over a defined period of time, in which different interventions are defined, sequenced, and documented in a way that facilitates communication and shared decision making. Stepped care allows for the most effective and least resource-intensive treatment to be provided by primary caregivers first, and then to move to more intensive or specialized services, but only to the extent that the complexity or severity of the patient's needs requires. This ensures that care is provided by the right person, at the right time, to the right patient. This link between primary and specialty care, based on clear and agreed-upon criteria, defines shared care. Finally, standardization of information enables effective and reliable communication within and between teams and with the family/patient, so that the patient moves smoothly through the various stages and levels of the care pathway. The project also includes a training program with an intensive online training core followed by weekly case discussion groups presented via videoconference by GPs with expert support over several months (e-clinic). In addition, we plan to implement a web-based intervention (Teacher Help, renamed ASSIST), which will allow teachers to learn about ADHD, and implement evidence-based strategies in their classrooms [17].

This model of care could address several challenges in the management of children with ADHD in urban China: scarcity of specialists but many more primary care providers, concentration of specialized skills, insufficient training, and lack of triage and referral system. In line with the Chinese guidelines for treating ADHD [12], the program also adopts a multimodal approach that combines educational, behavioural, and medical interventions. This paper is part of a large joint project between Canada and China with the goal of adapting and implementing the ADHD Shared Care Pathways program in the Chinese context. The current study aims to identify challenges and facilitators in adapting and implementing the ADHD Shared Care Program in a psychiatric care setting in Beijing, China.

## Materials and methods

### Sample and study setting

A purposive sampling method was used to select participants who were able to make a valuable contribution in understanding facilitators and barriers to implementation of the Shared Care Pathways program in Beijing. The Primary Investigator in Beijing (L.Y) used her understanding of the local contexts to select individuals who would be the most likely to be involved in implementing the project. Two semi-structured focus group sessions were conducted, the first with three expert specialists in the field of ADHD from Beijing Sixth Hospital (FG# 1) and the second with 4 general practitioners in Hai Dian Qu Wan Shou Lu Community Health Services Center (FG#2). The first group was composed of ADHD specialists, while the second focus group was composed of physicians without any specialized training in ADHD (see Table 1 for characteristics of the research participants).

**Table 1:** Characteristics of the research participants

Role	Extra Training on ADHD Management	Work Experience in years (In General/The Current Care Setting)	Usual Work Setting	FG	Qualification
Child psychiatrist	Yes	50/41	Tertiary Hospital	1	As an expert to guide the whole process of ADHD treatment and nursing, and responsible for outpatient treatment
Child psychiatrist	Yes	21/18	Tertiary Hospital	1	As an expert to guide the whole process of ADHD treatment and nursing, and responsible for outpatient treatment
Child psychiatrist	Yes	18/15	Tertiary Hospital	1	As an expert to guide the whole process of ADHD treatment and nursing, and responsible for outpatient treatment
Child psychiatrist	No	32/10	Secondary Hospital	2	Clinical treatment (assessment and consultation of ADHD students)
Director of psychiatric hospital	No	32/5	Secondary Hospital	2	Registered psychiatrist in China responsible for the management and coordination of their medical center
Psychiatrist	No	24/13	Community Center	2	Clinical treatment
Director of Community Center	No	11/3	Community Center	2	Registered psychiatrist in China responsible for the management and coordination of their medical center

### Data collection

Semi-structured focus groups were used to collect rich qualitative data for this research. Semi-structured interviews provide interviewers with the flexibility to explore themes that are particularly salient to participants. The Canadian researchers developed nine interview questions and specific probes to gather information on the current state of ADHD care as well as the facilitators and barriers to implement the Shared Care Pathways program in the local context (see the questions as supplemental material). The questions were developed based on the concepts of the Consolidated Framework for Implementation Research (CFIR) and were translated into Chinese. To ensure cross-cultural equivalence in translation, a 4-step translation process (forward translation, initial cross check, backward translation, and final cross-check) was adapted from the WHO guidelines for translation (see the current study’s translation guidelines as supplemental material). Because the data were collected prior to the implementation of the program in Beijing, the outline of the ADHD Shared Care Pathways program was sent to the research participants prior to the sessions. By means of sequential translation, the Canadian primary investigator (P.R) also explained the program in the beginning of the focus group sessions to ensure that everyone understand the program. Then, Chinese researchers (L.Y. & Q.Y.) asked the interview questions and facilitated discussions in the focus groups. Focus groups were done in-person and were digitally recorded (audio recording only) and transcribed as a means of ensuring accurate records and analyses.

### Data analysis

We analyzed the data according to the principles of Grounded Theory to identify barriers and facilitators for implementation of the project (Corbin and Strauss). The Grounded Theory is a rigorous and well-established approach which could be used by both Canadian and Chinese researchers. It allowed us (1) to immerse ourselves in the data, (2) to follow systematic, precise and clear coding procedures for transcripts, (3) to ensure that the analysis procedure was consistent across teams, and (4) to minimize the risk of including personal biases. Each focus group transcript was coded via open, axial, and selective coding. Data was first assessed using line-by-line open coding, in which significant units were labeled using descriptive codes. These codes were synthesized into categories based on the units’ common properties. Once we were satisfied with the open coding, researchers began the process of axial coding, whereby the relationships between the categories were determined through the researcher’s interpretive lens. In completing axial coding, the research team depicted the various relations between the categories. Lastly, researchers engaged in selective coding of the data, whereby the categories and their relationships will be interpreted to describe newly emerging themes. Using a grounded theory methodology provided conceptual clarity in the identification of barriers and facilitators for project implementation. Data analysis was completed using the NVivo qualitative software package (version 12).

### Data integrity

Several measures were utilized by the researcher to improve credibility and trustworthiness of the analysis, including: continuous comparative analysis, immersion in the topic of study, and persistent observation of the participants [18]. Dependability was adhered to through the consistent execution of study procedures. Furthermore, transferability was increased through providing information on the context in which the research was carried out, research participants, and methods [18]. To enhance confirmability, decisions made during the research process and emergence of the findings were reviewed in joint meetings with Canadian and Chinese researchers. As our objective was to adapt and implement a Canadian model of care in China, which have two very different cultures and health care organizations, we used a multi-coder approach to ensure methodological rigor. Two coders (members of the Canadian team) independently analyzed the data and came to a consensus on the results of the analysis. An additional researcher from the Chinese team (trained by the Canadian team) independently coded the transcripts, and all three researchers reached an agreement on the barriers and facilitators they identified.

## Results

Several themes emerged from the data analysis of the interviews. These themes included: (1) Social-level barriers and facilitators from the perspectives of clients and healthcare providers; (2) structural-level barriers and facilitators related to both internal and external organizational environments; and (3) intervention-related barriers and facilitators. The following sections describe these themes in detail and substantiate them using in-depth quotes taken verbatim from the participants within the study.

### Social-level barriers and facilitators

Items were coded as social-level if they assessed barriers/facilitators that represent the social context in which the organization is located. This theme has been emerged from two aspects: that of clients and that of the healthcare providers implementing the ADHD program. In what follows, we will first explain barriers and facilitators on behalf of clients and then, barriers and facilitators on behalf of healthcare providers.

### Social-level barriers on behalf of clients

Parents of children with ADHD seem to be unaccepting or distrustful of General Practitioners [GPs] working within basic-level hospitals when seeking help in treating their child’s mental health problems. This poor acceptance exists even when physicians within these hospitals are fully qualified and trained to treat such health problems. Instead, parents often actively seek the help of specialists within third-level hospitals. As one of the participants said: *“…, if basic doctors [GPs] are trained, they may gain some knowledge about ADHD… But the question is whether the patient would like to see them…?”*. In addition, several participants indicated that there exists an overall inadequate parents' education on the importance of the psychological health of their children “*ADHD itself is a disease with unknown etiology, that is to say, it must have something to do with parents' education and family activities. … Therefore, it is necessary to provide education [for parents] in this area”.* The research participants believed that this relatively low level of parental education also translates into a failure to screen for mental health problems in children. Finally, from a logistical standpoint, participants identified location and/or travel as a real barrier to accessing care. Some patients have to travel a long distance to see a specialist which is neither practical nor feasible for them. Whereas if the patients’ treatment was stable, they would rather to choose a place close to home to get the medication. The long queues that patients often face when seeking treatment were also seen as a barrier to accessing specialists: *“…there [at the basic level hospitals] is no need [for patients] to queue up [compare to a third level hospital] …*”.

Overall, with regards to social-level barriers that pertain to the Chinese clients, poor acceptance or distrust in the quality of physicians working at the basic-level hospital, low parental education, and burdensome logistical barriers were chiefly identified.

### Social-level facilitators on behalf of clients

In addition to the social-level barriers identified by participants within the focus groups, several key facilitators were discussed to enable implementation of this project.

To begin, participants indicated that the mandate and goals of the project are highly in line with the client demands within a Chinese healthcare context. Given that in the previous section, travel time and long wait times were identified barriers to accessing healthcare, participants indicated that this project would help address this issue. In particular, they believed that adopting a shared-care approach would incentivize clients to visit their GPs at basic level hospitals, which would then result in lower costs and faster patient turn-around. Mentioned during one of the focus groups: “*It's quite easy to see a doctor in our hospital [basic level hospital] … there is a certain discount for taking medications from basic hospitals…and there is no need to queue up … So, the patients may go there*”. Furthermore, participants believed this project would promote the education of parents, teachers, and classmates of children with ADHD, allowing for greater assistance for identification and management of children with ADHD. As one of the participants mentioned, *“… Especially in our country…. the prevalence of children’s problem is relatively high, their social functioning is damaged obviously, their families, classmates, teachers will also have such needs [increased awareness about ADHD]. … it is necessary to provide education in this area. One is education for parents and the other is education for teachers”*.

Developing a working partnership between GPs, specialists, teachers, and decision makers was recurrently mentioned as another facilitator during the focus groups. One of the research participants went so far as to indicate that: *“The biggest highlight of this project is that to integrate different parts together. Working together to get the task done instead of fighting alone individually. Personally, I think that is the biggest highlight”.*

A final facilitator to social-level implementation involves assessing the acceptability of the project from the patients' perspective: “*This project includes some interview, and will also ask about patients’ opinions, then … modifying the referral scheme [based on patients’ feedback]. Incorporating patients’ feedback can increase the chance of sustainability of the project”.*

To conclude, the client’s social-level facilitators comprise a mandate that matches their expectations, a belief that adopting a shared care approach would help with wait times and access to health services, promote education of parents and teachers of children with ADHD, develop a working partnership, and the ability to potentially assess of the acceptability of this project from the patients’ perspective.

### Social-level barriers on behalf of healthcare providers

In addition to the social-level barriers regarding clients, we identified themes related to barriers faced by healthcare providers. For example, participants mentioned in China, specialists are often the ones in charge of training GPs. However, given an already heavy workload and the resulting time constraints, some of these specialists are often unable or unwilling to provide the necessary mental health training for GPs*.* As one of the participants mentioned, *“Doctors in Third Grade hospitals cannot frequently go to basic hospitals for training. Especially when it is very likely to be a long-term thing*… *So, we’re …really intense with the time issue… How can I do it well, on the premise of not delaying my current work is a real challenge”.*

As mentioned by participants not all physicians are authorized to prescribe stimulant medications for patients with ADHD. As such, participants indicated that there is a need for a streamlined certification process to prescribe stimulants medication. Related to this issue is the uncertainty about the legal scopes of practice for different types of physicians. Several participants indicated that they had major concerns related to the risk of practicing beyond their scope of practice when treating patients with mental health challenges. For example: *“…For general practitioners, it is indeed a problem to practice beyond the scope, as a “specialist”. So, in this part, I think we need to get it approved legally”.*

Finally, the qualifications of physicians differ greatly from hospital to hospital, and from specialty to specialty. For example, as participants pointed out, a basic level hospital may be staffed only by undergraduates and GPs, while a third-level hospital may house several specialists: “*The qualifications of doctors at different levels are quite different… some doctors at the basic hospitals are undergraduate, some are even not”*. As such, participants believed that an additional barrier to implement the project is the heterogeneity of practitioners. Physicians who often come from different backgrounds raise a wide range of training needs in order to properly diagnose and manage patients. This situation makes the training of physicians in differing hospitals’ levels complex.

In summary, the social-level barriers faced by healthcare providers encompass issues related to training such as time constrain and heavy workload of specialists, barriers to proper prescription certifications, and the varying qualifications held by physicians within Level 1 to 3 hospitals which cause a wide range of training needs.

### Social-level facilitators on behalf of healthcare providers

Participants discussed at length the facilitators pertaining to healthcare providers in the implementation of this project. In general, they had a high degree of confidence and interest in the success of the project. For example, a participant indicated that: *“I give a score of 9* [out of 10 for success of the project*] …I can even give 9.5, based on history, and the scientific nature of the project, as well as social needs”.* Similarly, the project was seen to be highly in line with participants’ own values and goals, which may facilitate its successful implementation and high sustainability. Participants often indicated that they had expectations and believed that the project would greatly *“make improvement of medical treatment”* within a Chinese context.

### Structural-level barriers and facilitators

Items were coded as structural level if they assess barriers/facilitators that originate from the organizations in which the new ADHD program is being implemented (internal), or legal entities other than these organizations (external). In the following, we first explain the barriers and facilitators of the internal organization and then, the barriers and facilitators of the external organizations.

#### Internal organizational barriers

In speaking at length, participants identified several key barriers that they felt related to the internal organization of their respective hospitals and that could affect the implementation of the ADHD Shared Care Pathways program. Both GPs and specialists indicated that in order for this project to be successful, barriers related to an absence of support on behalf of leaders at hospitals from all levels would need to be addressed. For example, participants indicated that a lack of internal policies that would ultimately support this new program could be a barrier to implementation: *“…and, we also need their support… Whether the leaders of these hospitals support this kind of work also matters”,,,,,* “*To ensure we can do our work well, we must have policies … so that we can have full guarantee for other stuff”.*

On a related note, an inadequate financial and human resources (technical and administrative staff) would greatly hinder implementation: *“we should have the resources from our hospital. Now in fact, your [referring to the tertiary level hospitals] burden is very heavy. If you do anything else, you have to make alternatives for the out-patient clinic. we should have the resources from our hospital… I don't know what the situation of the basic hospital is…Do they have enough personnel?” … “…we must have financial expenditures…”.*

Finally, participants indicated that a lack of an adequate referral system, which would allow patients to be referred from primary to specialized hospitals, and back to the primary hospital, was a definite barrier: *“The current referral system is still very imperfect…the problem is that the patients in Third Grade hospital are not transferred here [the basic level hospital] …”.* Therefore, it is necessary to design a proper referral system between the hospitals where the project will be implemented.

To conclude, internal organizational barriers identified by participants in both focus groups included an insufficient organizational support, a lack of financial and human resources, and an unclear or non-existent patient referral system.

#### Internal organizational facilitators

The compatibility of the project with the organizational culture present within many of the participating hospitals was mentioned as a major facilitator: “*According to the history, it [the designated hospital's culture and values] is matched [with the current project]”.* One of the participants explained that the culture of their hospital supports innovation, and that is the reason for this compatibility. As was elaborated by another participant: *“… I think there should be no problem in the aspect of culture… In fact, in terms of our values and culture, we serve the general population and definitely can provide such services for children.”*

Some of the hospitals that were represented by the focus group participants have ongoing connections within their communities, including their local school systems, which increases the chance of success in implementation of the project. Such connections were indicated by almost all participants: “*In fact, I think, especially in psychiatry, we have done some services in schools, including primary and secondary schools, and we also have certain communication with schools.”*

#### External organizational barriers

External organizational barriers were identified in the form of an absence of support on behalf of municipal and state governments. Namely, participants indicated that inadequate government-level involvement would ultimately hinder the implementation of this project (or any others like it): *“Some of the slogans we have been spoken for 10 years did not solve the problem, and some of the medical staff have changed careers…That's why I emphasized that in this process, we should let the decision-makers [at the governmental level] participate and let them have a look at the actual situation. Otherwise, doctors in the top hospitals are too burdened to think or study at all”.*

#### External organizational facilitators

Many external organization facilitators were discussed by the participants from both focus groups. For example, participants indicated that the mandate of the project was highly compatible with the needs of current medical triage practices and the current direction of medical care organization, particularly in Beijing. One of the participants mentioned the project was highly compatible with the *“management system of the Haidian district”*.

Participants named a hospital in Beijing that has been relatively successful in the grading diagnosis, and treatment of patients by expert medical teams: *“I know they have expert teams …such as for depression, ADHD, *etc.*… the number of outpatients of their attending doctors is relatively large… Top experts have to be recommended level by level…”.* Since an appropriate patient referral system was deemed as necessary for the current project, the use of the aforementioned hospital’s patient referral model was suggested as a possible facilitator.

### Intervention-related barriers and facilitators

Items were coded as intervention-level if they assess barriers/facilitators that were aspects of the proposed ADHD Shared Care Pathways program to be implemented. In the following, first the barriers and then the facilitators related to this theme have been explained.

#### Intervention-related barriers

The participants indicated the need for a detailed outline of the proposed program. Herein, participants wanted details on all internal procedures, especially as they pertain to the referral system. One of the participants asked: *“For example, this kid has been treated, but he has relapsed later. Under this situation… should we tell the child to go to the specialist directly or wait for the specialist to come over?”*. Another participant mentioned: *“But we should discuss what you [GPs] come to us [Specialist] [to receive training] for, and what tasks you [GPs] need to solve after you go back [to your hospital]”.*

Related to this aforementioned barrier, participants indicated that the program outline should consider the reality or context of China’s current healthcare setting. For example, this program should take into account the limited availability of specialists who can provide training for GPs: “*I'm also thinking about the time issue if I'm going to do something really detailed. Because there are so many things in hand…”.*

The final barrier pertains to the current design of the training program. One of the participants felt that online training may not be sufficient for their needs: “*In the past, there was such [online] training because of the inconvenient transportation. But the effect of the training was not very good”.* The failure to provide additional in-person training could hinder the learning of those involved in the project.

Overall, intervention-related barriers encompassed limited detail in the program outline, unavailability of specialists who can provide training, and the possibility that online training may be inadequate.

#### Intervention-related facilitators

A significant number of facilitators that were identified within this study pertain to the intervention. To begin, participants described the rigorous design of the project very acceptable for stakeholders in China. As was elucidated: *“the design of this research is very rigorous…there are very rigorous qualitative research methods… Through such a model, once established, it must be very scientific, effective and generalizable”.*

Participants indicated that a series of exams, designed for the online course, would increase the overall effectiveness of the training: *“We can add in exams. So online training plus exams, some tasks and so on. This will be better”.* Further related to the training aspect of this intervention, they felt that adequate time should be allocated to specialists to prepare the courses (in cases where they train GPs), and that dividing the training tasks among differing specialists would reduce the overall burden associated with meeting training goals.

Another suggested facilitator to the project would be ensuring that the tools used to assess the project are short and/or simple and that the project is completed in small, adaptive parts; much like a pilot: “*But I still have some ideas. Don't do too much at one time, because I think starting from one point, then after you have accumulated enough experience and made some improvement, you can slowly move on”*. Participants mentioned having a clearly stated end-goal for the project would also contribute to the sustainability and overall longevity of the project.

In conclusion, the intervention-related facilitators that were discussed by participants included the strong scientific foundations that make up this study, the allocation of adequate time to prepare training, the use of training tests, ensuring that the questionnaires/assessments used during the study do not overwhelm participants, and having a clearly-stated end goal for the project.

## Discussion

The purpose of this study was to assess the challenges and facilitators in adapting and implementing an ADHD Shared Care Pathways program developed in Canada in a Chinese context, specifically in a mental health setting in Beijing. In this study, we engaged healthcare providers (specialists and GPs) who work at designated hospitals where the project will be implemented in future. Understanding the challenges and facilitators from the viewpoint of those who are likely to be able to use the results of this research in their practice can enhance research uptake and project sustainability in a similar context [19].

In the current study we identified three main themes: (1) Social-level barriers and facilitators faced by healthcare users and healthcare providers; (2) structural-level barriers and facilitators related to both internal and external organizational environments; and (3) barriers and facilitators specifically related to our intervention. Understandably, these themes are a result of the differences between Chinese and Canadian approaches and therefore can both hinder or facilitate the implementation of the shared-care approach to ADHD management.

### Socio-cultural barriers

As a preface, our analysis of the socio-cultural barriers in implementing a Canadian shared-care approach to ADHD treatment in China included parental low level of education, mistrust in the Chinese primary healthcare system, long travel times due to the geographic layout of Beijing hospitals, and the low perceived competence of healthcare practitioners. These barriers may partly explain why ADHD is detected in some children and not in others in a timely manner. Low parental education about mental health issues, which in turn translated into a failure to engage in treatment-seeking behaviours, was one of the main socio-cultural barriers identified by our participants to program implementation. This issue is exacerbated by the patients’ mistrust in the quality of GPs and their preference to consult specialists for most of their health needs. Given Beijing’s geographic layout, many patients must travel long distances to visit these specialists who are in few numbers and only available in a few third-level hospitals. The cumulative effect of this ultimately results in longer wait times as more and more patients opt for this option. On the other hand, our research participants mentioned that many GPs do not consider themselves competent enough to be in charge of the diagnosis and treatment of children with ADHD. As a result, they refer the patients to the third-level hospitals which worsen the waiting time to access specialists. According to participants, the varying qualifications of GPs in China may account for this perceived poor competence, as not all of them receive the same level of medical education and are licensed to prescribe stimulant medication.

Clients’ treatment-seeking behaviours is highly important for timely recognition and quality management of ADHD [20]. Results of our study attribute this failure to engage in care-seeking to parents’ insufficient knowledge about mental health issues and mistrust in health services provided by GPs at the first and second level hospitals. Such findings highlight the need for community education regarding indicators, proper treatment, and available healthcare services for ADHD. To date, the source of parents’ lack of trust has not been studied in China. However, it may be rooted in the lower level of education of physicians in first- and second-level hospitals. According to the China Health Statistics [13], 55.2% of licensed GPs in cities and 82.4% in rural areas did not hold a bachelor’s degree from a medical university. Patients may expect physicians to be able to meet all of their health needs, but because this expectation is excessive for the capabilities of primary care physicians, it can cause dissatisfaction and mistrust when it is not met. Parents’ distrust in health services has also been reported in Western countries as a barrier to ADHD treatment-seeking behavior [21]. However, the societal perception of ADHD can fuel this mistrust. For example, the claim that ADHD does not exist but is the manifestation of many different disorders that should be treated separately and that grouping them together under the single diagnosis of ADHD has triggered an unwarranted epidemic of stimulants is likely to contribute to the public's distrust of medical treatment for ADHD [22]. Other parents perceive ADHD as a means of increasing profits for the pharmaceutical industry and its affiliates, and a series of class-actions federal lawsuits were filled in the US alleging that the American Psychiatric Association was aiding and abetting an "inappropriate use" of stimulant medication [23, 24]. While such actions may undermine parental confidence in the validity of ADHD diagnosis and treatment, the majority of pediatricians in the United States reported in both 2004 and 2013 that they lack confidence and training in the use of psychotropic medications [25]. In the UK, as in many other Western countries, the general practitioner is the gatekeeper for referral to a specialist, and their inability or unwillingness to identify ADHD is the main barrier to treatment [26]. In conclusion, in both health systems, insufficient training of general practitioners thus contributes to delayed access to care, either because wary parents bypass the general practitioner or because the general practitioner consulted is poorly trained to make a diagnosis or referral. To varying degrees, as we estimated a ratio of qualified specialists to the child population of 1:40,000 in Canada and 1:400,000 in China, the shortage of specialists exacerbates difficulties in accessing care.

### Socio-cultural facilitators

Our research participants believed adopting the Shared Care Pathways program would help overcoming some of the aforementioned socio-cultural barriers. Through this program, GPs will be trained to provide screening and initial ADHD treatment. Training GPs not only increase their own confidence in managing ADHD, but also enhances parents’ trust in the services that GPs provide. Training ultimately promotes patients’ access to health services and initiation of timely treatment as GPs are more accessible than specialists. However, if a patient’s level of care exceeds that of routine care, the GP will be able to receive a consultation form specialist and refer patients to that specialist if necessary, and thus ultimately facilitate access to more specialized care. Participants of our research also mentioned this program will facilitate community education by providing training programs for parents and teachers about ADHD through schools. On the other hand, they believed the Shared Care Pathways program is consistent with healthcare providers’ own values and goals. Therefore, they would be very interested to implement the program at their care settings. According to the Consolidated Framework for Implementing Research (CFIR) [27], effective implementation can be anticipated by the extent to which the project goal matches the needs and values of patients and care providers. The more alignment they perceive between the meaning they attach to the new approach and their needs, the more readily they accept the proposed approach and subsequently contribute to its successful implementation [27].

### Structural-level barriers

Our analysis revealed several organizational barriers that may hinder the implementation of a shared-care approach to ADHD treatment in China. From a hospital-specific standpoint, the absence of support from leaders, limited resources, and an inappropriate patient referral system were identified as chief concerns. An overarching and exacerbating component may be the limited support from municipal and state governments for mental healthcare in China. Therefore, our research participants believed that implementing the new approach requires internal and governmental policies, resource allocation, and technical support, especially to establish effective referral systems within the hospitals.

Leadership commitment in terms of interest and engagement in a new approach leads to a better implementation climate and subsequently implementation effectiveness [28, 29]. High and middle-level hospital leaders play a critical role in providing internal policies, negotiating resources, creating a climate to support employees’ learning, and fostering collaboration across teams [30, 31]. According to Gershon et al. (2004), the compatibility of the new intervention with the organization goal as well as the engagement of leaders in the decision-making process regarding implementation increase the likelihood of their support [31]. Leadership support is crucial to develop capacities, especially those needed for the specific implementation of this project. The innovation-specific capacities should be expanded at the individual level (motivation and skills) and at the organizational level (human, technical, physical, and financial resources) [32, 33]. Building specific capacities will allow the project to continue without further dependence on external support. In the long run, a national support policy and guidelines are necessary to establish all the essential resources to improve the efficiency and sustainability of new implementations [27].

### Structural-level facilitators

As in response to the concerns just described, research participants believed that the compatibility of the project with the culture of the designated hospitals (e.g., supporting new and innovative approaches) and the current direction of local government can facilitate the dialogue with them to overcome the structural barriers just discussed. In 2016, Peking University Sixth Hospital signed an agreement with the Haidian Mental Health Hospital and associated community hospitals to develop a partnership to improve the management of mental health services in lower-level hospitals. Under this agreement, these hospitals will work together to provide logistic support (e.g., space, salary and training) and implement referral policies between primary and specialized services. The shared care pathways program fits within this agreement as if it were arising from it, as it promotes a collaborative approach between different level hospitals. Research participants emphasized that the designated hospitals also have a good connection with schools, which facilitates the implementation of the program’s web-based intervention in the schools. Cosmopolitanism is an important factor that impacts the success of implementation [27]. Organizations that establish external networking are more prone to implement new practices quickly [34]. In highly cosmopolitan organizations, peer pressure (i.e., emulation among organizations) has a positive association with implementation success, as demonstrated by the example of another hospital in Beijing that developed a referral system that could serve as a model in this project.

### Intervention-level barriers

In general, two main related factors emerged that require special attention: the issue of accountability of the different stakeholders in the project, and the issue of stakeholder competence development. These two factors are related as participants can be held accountable for their role only to the degree that they have the competencies to fulfill it.

To begin, the participants identified as a barrier the need for a more detailed description of the proposed program, particularly with respect to the referral system and the roles of different levels of physicians. In this context, accountability means that everyone understands and accepts their assigned role, and feels responsible for accomplishing their designated tasks [35]. The detailed assignment of accountabilities (i.e., who does what and when) has been identified as extremely helpful for the research uptake [36]. In keeping with this principle of accountability, a care pathway is a set of discrete but related interventions for which it is clear who is responsible for what [37]. However, within a care pathway, how the care process is organized, and the respective responsibilities are defined, depends on the existing guidelines and the level of agreement between team members. Therefore, the different professionals involved in the care pathway need to reach a solid agreement on the details of the different steps, and on how to share responsibilities. This agreement can only be reached through a consensus process, and the details of responsibilities can only be defined after the adoption of an overall plan and initial training, which allows team members to define their more advanced training needs, and gain confidence that they are capable of fulfilling their assigned role. Above all, the Canadian team wanted to avoid the project being perceived as a "copy and paste" of a foreign model. On the other hand, identifying that we had given little detail about responsibilities as a barrier showed that participants fully understood that the key to an effective care pathway is that responsibilities must be clear and transparent.

Another identified barrier was the possibility that our project would not sufficiently take into account the reality of the Chinese healthcare system, and in particular the limited number of specialists available in each hospital. Because of their heavy clinical responsibilities, specialists have very little time available to train GPs. No one disputes the need for training, however, as the suboptimal training of many primary care providers was clearly identified as a social barrier. Improving the quality of training was the first recommendation of a recent synthesis paper on the quality of primary care in China. This training should enable them to achieve an adequate level of clinical skills and prepare them to work in interprofessional teams [38]. While one way to address this limited availability is to use self-paced online trainings, this solution itself faces the identified barrier that such online trainings were felt to be inadequate for developing the skills and knowledge necessary for successful implementation of our intervention. It is true that the common ‘gold standard’ for training health care providers is in-person workshops, supplemented with manuals and clinical supervision [39]. However, a meta-analysis concluded that online methods may be as effective as alternative methods for training clinicians for the outcomes of knowledge and clinical behaviour [40]. In China, a teacher-focused tradition created an expectation that the learner must acquire a legitimate knowledge directly from teachers rather than taking responsibility for their own training in a more interactive, self-paced, web-based pedagogy [41]. While in-person training is certainly valued, there is also a desire for innovative alternative practices, which are further enhanced by restrictions on travel and meetings with the current COVID-19 crisis.

### Intervention level facilitator

In terms of intervention-specific facilitators, participants mentioned that the project had a strong scientific basis. Similarly, having a clearly defined endpoint and proposing clinical assessment tools that would not overwhelm participants were also seen as facilitators. Finally, allowing sufficient time for specialists to prepare materials for training GPs and evaluating the effectiveness of the training were also identified as factors facilitating the success of the training phase of the project.

In China, most of the research stops at publication, but the awareness is growing that the next step is to apply the research to solve real-life problems [42]. The use of evidence is related to the level of scientific literacy of health care decision makers [43] and trust in the quality of research [44]. Policy-makers use information they can trust. A project with rigorous methodologies and scientific references, especially if supported by a trusted international organization, is more likely to be adopted [35]. This international research project, conducted under the auspices of the Global Alliance for Chronic Diseases (GACD) and funded by China and Canada, met the conditions to be seen as scientifically sound. Along the same lines, having clear objectives and proposing well-structured research materials are also facilitators of successful implementation of the intervention [45]. Three studies conducted in Australia, Canada, and Ireland which explore the factors influencing research utilisation in each country highlighted the importance of clear articulation of the research with the knowledge users [46–48]. Compatible with the results of our study, these three studies identified having clear goals and providing well-structured research materials as intervention-related facilitators to success of implementation.

As outlined by the CFIR (2009), the level of resources that are dedicated to implementing a project such as time, funds, and staff reveals the degree of the feasibility of the innovation [27]. Along with the aforementioned factors, the stakeholders’ willingness for change and to provide the conditions for the change (e.g., time for preparation, etc.) plays a crucial role in feasibility of innovation.

Identified barriers and facilitators in this study can, at their roots, be attributed to the differing socio-political and cultural contexts unique to the Chinese healthcare system. Although both Canada (the country that the program was originally developed in) and China have relatively strong and responsive healthcare systems, their core makeup can be very different. It was important to understand the healthcare landscape in China in order to properly situate our intervention within its greater context.

## Limitations

Limitations of the study relate to the relatively low number of participants in this research which may reduce the variety of perspectives. The number of participants was dictated by the small number of ADHD specialists and the limited availability of specialists and general practitioners in tertiary, secondary, and community health settings.

Interviewing service users themselves would have been useful but proved impossible at this early stage. Our Chinese stakeholders felt that service users would not come and participate effectively in the focus group discussions because they do not yet trust general practitioners, but that they could be involved in the evaluation of the project once the referral system and training were in place.

## Conclusion

We conducted a qualitative study to systematically examine the factors influencing the implementation of the ADHD Shared Care Pathways program in a Chinese context. We learned that there are a number of factors which could hinder or improve the uptake of the program in China, including issues relating to the program itself (intervention factors), aspects relating to clients and healthcare providers (socio-cultural factors), and the context where the knowledge will be used (structural factors). The identified barriers and facilitators contribute to the adaptation of the program to fit local circumstances and eventually increase uptake and sustained use of the program. Two of the major barriers in China that may interfere with the implementation of the program are first, varying qualifications held by physicians within differing hospitals which suggest a wide range of training needs regarding ADHD management, and second, lack of appropriate patient referral systems between different levels of hospitals.

Our chosen approach allows us to slowly and dynamically implement a shared-care approach within a very different context in which it was first developed. Instead of inserting ourselves within a vastly different medical system and dictating changes, our adaptive approach allows for the proper identification and allocation of resources and fosters investment in the goals of the project. We are continuing our collaboration with the local stakeholders to overcome other identified barriers in order to enhance the possibility of successful implementation. An effective implementation improves timely and quality ADHD management in children and adolescents and could also be a model for many other similar conditions in China or other developing countries.

## Supplementary Information

**Additional file 1:** 

**Additional file 2:** 

## Data Availability

The datasets generated during and/or analysed during the current study are available from the corresponding author on reasonable request.

## References

[1] Tong L, Shi H, Zang J (2013). Prevalence of ADHD in children of China: a systematic review and meta analysis. Chin J Public Health.

[2] Agnew-Blais JC, Polanczyk GV, Danese A, Wertz J, Moffitt TE, Arseneault L (2016). Evaluation of the Persistence, Remission, and Emergence of Attention-Deficit/Hyperactivity Disorder in Young Adulthood. JAMA Psychiat.

[3] Biederman J, Petty CR, Dolan C, Hughes S, Mick E, Monuteaux MC (2008). The long-term longitudinal course of oppositional defiant disorder and conduct disorder in ADHD boys: findings from a controlled 10-year prospective longitudinal follow-up study. Psychol Med.

[4] Sobanski E, Banaschewski T, Asherson P, Buitelaar J, Chen W, Franke B (2010). Emotional lability in children and adolescents with attention deficit/hyperactivity disorder (ADHD): clinical correlates and familial prevalence. J Child Psychol Psychiatry.

[5] Biederman J, Petty CR, Fried R, Kaiser R, Dolan CR, Schoenfeld S (2008). Educational and occupational underattainment in adults with attention-deficit/hyperactivity disorder: a controlled study. J Clin Psychiatry.

[6] Dalsgaard S, Ostergaard SD, Leckman JF, Mortensen PB, Pedersen MG (2015). Mortality in children, adolescents, and adults with attention deficit hyperactivity disorder: a nationwide cohort study. Lancet.

[7] Biederman J, Wilens TE, Mick E, Faraone SV, Spencer T (1998). Does attention-deficit hyperactivity disorder impact the developmental course of drug and alcohol abuse and dependence?. Biol Psychiatry.

[8] Kates N, Mazowita G, Lemire F, Jayabarathan A, Bland R, Selby P (2011). The evolution of collaborative mental health care in Canada: A shared vision for the future. The Canadian Journal of Psychiatry / La Revue canadienne de psychiatrie.

[9] Sonuga-Barke EJ, Brandeis D, Cortese S, Daley D, Ferrin M, Holtmann M (2013). Nonpharmacological interventions for ADHD: systematic review and meta-analyses of randomized controlled trials of dietary and psychological treatments. Am J Psychiatry.

[10] National Collaborating Centre for Mental H. National Institute for Health and Clinical Excellence: Guidance. Attention Deficit Hyperactivity Disorder: Diagnosis and Management of ADHD in Children, Young People and Adults. Leicester (UK): British Psychological Society (UK) Copyright © 2009, The British Psychological Society & The Royal College of Psychiatrists.; 2009.

[11] Pliszka S (2007). Practice parameter for the assessment and treatment of children and adolescents with attention-deficit/hyperactivity disorder. J Am Acad Child Adolesc Psychiatry.

[12] Zheng YL, J. Chinese Guidelines for the Prevention and Treatment of Attention Deficit Hyperactivity Disorder. 2 ed. Beijing: Chinese Medical Multimedia Press; 2015.

[13] Milcent C (2018). Healthcare Reform in China: From Violence To Digital Healthcare.

[14] Anand S, Fan VY, Zhang J, Zhang L, Ke Y, Dong Z (2008). China's human resources for health: quantity, quality, and distribution. Lancet.

[15] Canadian Psychiatric Association and The College of Family Physicians in Canada. Shared mental health care in Canada: Current status, commentary, and recommendations. A report of the collaborative working group on shared mental health care.2000 March 27, 2017. Available from: http://www.cfpc.ca/uploadedFiles/Resources/Resource_Items/Health_Professionals/Shared_mental_health_care.pdf.

[16] Bower P, Garralda E, Kramer T, Harrington R, Sibbald B (2001). The treatment of child and adolescent mental health problems in primary care: a systematic review. Fam Pract.

[17] Corkum  P , Elik  N , Blotnicky-Gallant  PAC , McGonnell  M , McGrath  PJ (2015). Web-Based Intervention for Teachers of Elementary Students With ADHD. Journal of Attention Disorders.

[18] DiMillo J, Samson A, Thériault A, Lowry S, Corsini L, Verma S (2013). Living with the BRCA genetic mutation: an uncertain conclusion to an unending process. Psychol Health Med.

[19] Macaulay AC, Jagosh J, Seller R, Henderson J, Cargo M, Greenhalgh T (2011). Assessing the benefits of participatory research: a rationale for a realist review. Glob Health Promot.

[20] Wright N, Moldavsky M, Schneider J, Chakrabarti I, Coates J, Daley D (2015). Practitioner Review: Pathways to care for ADHD - a systematic review of barriers and facilitators. J Child Psychol Psychiatry.

[21] Hamed AM, Kauer AJ, Stevens HE. Why the Diagnosis of Attention Deficit Hyperactivity Disorder Matters. Front Psychiatry. 2015;6:168-. 10.3389/fpsyt.2015.00168 PMC4659921 26635643

[22] Saul R. ADHD Does Not Exist: The Truth About Attention Deficit and Hyperactivity Disorder. 1 ed. New York, NY: Harper Wave; 2014.

[23] Hervey-Jumper H, Douyon K, Franco KN (2006). Deficits in diagnosis, treatment and continuity of care in African-American children and adolescents with ADHD. J Natl Med Assoc.

[24] Ellison P. Science over cynicism. Children and Adults with Attention-Deficit Hyperactivity (CHADD). . Attention Magazine. 2003;1:27–33.

[25] Horwitz SM, Storfer-Isser A, Kerker BD, Szilagyi M, Garner A, O'Connor KG (2015). Barriers to the Identification and Management of Psychosocial Problems: Changes From 2004 to 2013. Acad Pediatr.

[26] Sayal K, Taylor E, Beecham J, Byrne P (2002). Pathways to care in children at risk of attention-deficit hyperactivity disorder. Br J Psychiatry.

[27] Damschroder LJ, Aron DC, Keith RE, Kirsh SR, Alexander JA, Lowery JC (2009). Fostering implementation of health services research findings into practice: a consolidated framework for advancing implementation science. Implement Sci.

[28] Klein KJ, Conn AB, Sorra JS (2001). Implementing computerized technology: an organizational analysis. J Appl Psychol.

[29] Lukas CV, Holmes SK, Cohen AB, Restuccia J, Cramer IE, Shwartz M (2007). Transformational change in health care systems: an organizational model. Health Care Manage Rev.

[30] Leeman J, Baernholdt M, Sandelowski M (2007). Developing a theory-based taxonomy of methods for implementing change in practice. J Adv Nurs.

[31] Gershon RR, Stone PW, Bakken S, Larson E (2004). Measurement of organizational culture and climate in healthcare. J Nurs Adm.

[32] McKay VR, Dolcini MM, Catania JA (2017). Impact of Human Resources on Implementing an Evidence-Based HIV Prevention Intervention. AIDS Behav.

[33] Flaspohler P, Duffy J, Wandersman A, Stillman L, Maras MA (2008). Unpacking prevention capacity: an intersection of research-to-practice models and community-centered models. Am J Community Psychol.

[34] Greenhalgh T, Robert G, Macfarlane F, Bate P, Kyriakidou O (2004). Diffusion of innovations in service organizations: systematic review and recommendations. Milbank Q.

[35] Ferlie E (2016). The Oxford Handbook of Health Care Management.

[36] Albert MA, Fretheim A, Maiga D (2007). Factors influencing the utilization of research findings by health policy-makers in a developing country: the selection of Mali's essential medicines. Health Res Policy Syst.

[37] Vanhaecht K (2007). The impact of Clinical Pathways on the organisation of care processes.

[38] Li X, Krumholz HM, Yip W, Cheng KK, De Maeseneer J, Meng Q (2020). Quality of primary health care in China: challenges and recommendations. The Lancet.

[39] Beidas RS, Kendall PC (2010). Training Therapists in Evidence-Based Practice: A Critical Review of Studies From a Systems-Contextual Perspective. Clin Psychol (New York).

[40] Richmond H, Copsey B, Hall AM, Davies D, Lamb SE (2017). A systematic review and meta-analysis of online versus alternative methods for training licensed health care professionals to deliver clinical interventions. BMC Med Educ.

[41] Barratt-Pugh L, Zhao F, Zhang Z, Wang S (2019). Exploring current Chinese higher education pedagogic tensions through an activity theory lens. High Educ.

[42] Jiang F, Zhang J, Shen X (2013). Towards evidence-based public health policy in China. The Lancet.

[43] Zdunek K, Alexander D, Schröder-Bäck P, Rigby M, Blair M (2021). Factors influencing the uptake of evidence in child health policy-making: results of a survey among 23 European countries. Health Research Policy and Systems.

[44] Albert MA, Fretheim A, Maïga D (2007). Factors influencing the utilization of research findings by health policy-makers in a developing country: the selection of Mali's essential medicines. Health Research Policy and Systems.

[45] Buckley H, Tonmyr L, Lewig K, Jack S (2014). Factors Influencing the Uptake of Research Evidence in Child Welfare: A Synthesis of Findings from Australia. Canada and Ireland Child Abuse Review.

[46] Jack S, Dobbins M, Tonmyr L, Dudding P, Brooks S, Kennedy B (2010). Research evidence utilization in policy development by child welfare administrators. Child Welfare.

[47] Arney F, Bromfield L, Lewig K, Holzer P (2009). Integrating strategies for delivering evidence-informed practice. Evidence & Policy: A Journal of Research, Debate and Practice.

[48] Buckley H. Whelan S. Putting Research Evidence to Work: Key Issues for Research Utilisation in Irish Children's Services. Dublin: Children Acts Advisory Board 2009 2009.

