# Correction: Implementing a Canadian shared-care ADHD program in Beijing: Barriers and facilitators to consider prior to start-up

**DOI:** 10.1186/s12888-022-04361-9

**Published:** 2022-11-14

**Authors:** Sayna Bahraini, Alexander R. Maisonneuve, Yirong Liu, André Samson, Qian Ying, Fei Li, Li Yang, Philippe Robaey

**Affiliations:** 1 Children’s Hospital of Eastern Ontario, University of Ottawa, Ottawa, Canada; 2 Peking University Sixth Hospital, Peking University Institute of Mental Health, National Clinical Research Center for Mental Disorders (Peking University Sixth Hospital), NHC Key Laboratory of Mental Health (Peking University), Beijing, 100191 China; 3 Faculty of Education, University of Ottawa, Ottawa, Canada; 4 Developmental and Behavioral Pediatric Department and Child Primary Care Department, Ministry of Education, Shanghai Key Lab for Children’s Environmental Health, Xinhua Hospital, Shanghai Jiaotong University School of Medicine, Shanghai, China

**Correction: BMC Psychiatry 22, 321 (2022)**

**https://doi.org/10.1186/s12888-022-03955-7**

Following publication of the original article [1], the authors identified an error in the author name of Alexander R. Maisonneuve.

The incorrect author name is: Alexander R. Maisoneuve

The correct author name is: Alexander R. Maisonneuve

The author group﻿ has been updated above and the original article [1] has been corrected.

## References

[1] Bahraini (2022). Implementing a Canadian shared-care ADHD program in Beijing: Barriers and facilitators to consider prior to start-up. BMC Psychiatry.

