# Supplementary material for: Implementing a Canadian shared-care ADHD program in Beijing: Barriers and facilitators to consider prior to start-up
Source: BMC Psychiatry. 2022 May 5;22:321. doi: 10.1186/s12888-022-03955-7 (PMC9069949; doi:10.1186/s12888-022-03955-7)
Supplement: Supplementary file 1 — Additional file 1: [file 12888_2022_3955_MOESM1_ESM.docx]

FG interview questions :

1. What is your perception of this new ADHD program with regard to evidence strength and scientific quality?
2. What kinds of changes do you think you will need to make to implement the new ADHD program, so it will work effectively in your setting? Do you think you will be able to make these changes in your setting?
3. How complex does the new ADHD program looks like for you, when it comes to duration, scope, number of steps, type of professionals and organizations involved?
4. What kind of training is needed to successfully implement the project?
5. How would you describe the culture (general beliefs, values, assumptions that people embrace) of your organization? Of your own setting or unit? How do you think your organization's culture will affect the implementation of the new ADHD program?
6. Is there a strong need for this new ADHD program?
7. Do you expect to have sufficient resources (including money, training, education, physical space, and time) to implement and administer the new ADHD program? [If Yes] What resources are you counting on?
8. How confident are you that you will be able to successfully provide changes or take actions to achieve the implementation goals of the new ADHD program? What gives you that level of confidence (or lack of confidence)?
9. What do you prefer about the new ADHD program?
