# Supplementary material for: Implementing a Canadian shared-care ADHD program in Beijing: Barriers and facilitators to consider prior to start-up
Source: BMC Psychiatry. 2022 May 5;22:321. doi: 10.1186/s12888-022-03955-7 (PMC9069949; doi:10.1186/s12888-022-03955-7)
Supplement: Supplementary file 2 — Additional file 2: [file 12888_2022_3955_MOESM2_ESM.docx]

**Translation Guideline**

Dear Translators,

The objective of the current study is to adapt a Canadian shared care model for Attention-Deficit/Hyperactivity Disorder (ADHD) to Chinese hospitals. To achieve this goal, we need to translate research materials such as questionnaires, interview questions, and codebooks.

**Process of Translation of the Research Materials:**

The aim of this process is to achieve Chinese or English research materials that are conceptually equivalent in each of the target countries/cultures. That is, the English and Chinese research materials such as a questionnaire should be equally natural and acceptable and should practically perform in the same way. The focus is on cross-cultural and conceptual, rather than on linguistic/literal equivalence. A well-established method to achieve this goal is to use forward-translations and back-translations. Implementation of this method includes the following steps:

- Forward-translation
- Initial Cross-check
- Back-translation
- Final Cross-check

**1) Forward-translation**

The translator should be knowledgeable of the second language culture, but his/her mother tongue should be the primary language of the target culture (i.e., a Chinese native speaker preferably translates English materials to Chinese)

We emphasize conceptual rather than literal translations and stress the need to use natural and acceptable language for the broadest audience. The following general guidelines should be considered in this process:

- Translators should always aim at the conceptual equivalent of a word or phrase, not a word-for-word translation, i.e. not a literal translation. The translator should consider the definition of the original term and attempt to translate it in the most relevant way.
- Translators should strive to be simple, clear and concise in formulating a question. Fewer words are better. Long sentences with many clauses should be avoided.
- The target language should aim for the most common audience. Translators should avoid addressing professional audiences such as those in medicine or any other professional group. They should consider the typical respondent for the instrument being translated and what the respondent will understand when she/he hears the question.

Translators should avoid the use of any jargon. For example, they should not use technical terms that cannot be understood clearly; and colloquialism, idioms or vernacular terms that cannot be understood by common people in everyday life.

- Translators should consider issues of gender and age applicability and avoid any terms that might be considered offensive to the target population.

### 2) Initial Cross-Check

An expert in the mental health field (Preferably, bilingual in English and Chinese) will cross-check the forward translation. The goal in this step is to identify and resolve the inadequate expressions/concepts of the translation, as well as any discrepancies between the forward translation and the existing or comparable previous versions of the questions if any. The expert may question some words or expressions and suggest alternatives. The principal investigator or the research advisor will provide the expert any materials that can help her/him to be consistent with previous translations. The expert will be in touch with the principal investigator and the forward translator.

The result of this process will produce a complete translated version of the research material.

### 3) Back-translation

Using the same approach as that outlined in the first step, the research material will then be translated back to the original language that it was initially developed by an independent translator, whose mother tongue is the target culture and who has no knowledge of the research material.

As in the initial translation, emphasis in the back-translation should be on conceptual and cultural equivalence and not linguistic equivalence.

**4) Final Cross-check**

The forward translator will compare the original research material, forward translation, and back translation. Discrepancies should be discussed with an expert in the mental health field and the back-translator. Further work should be iterated as many times as needed until a satisfactory version is reached. The forward translator will prepare the final version after resolving conflicts.

**Translators’ role throughout the translation process:**

Translators responsibilities include reading, thoroughly understanding, translating the given materials, and proofreading finished pieces of work. To be successful in this role, the translators should have a keen eye for detail. Ultimately, they will provide ready-to-use translated content that meets the internal needs of the project.

**Readability of the document**: Translators should ensure that the information is translated at a reading level appropriate for general population. We have determined a general grade 8 reading level using Flesch–Kincaid.

**How to check readability of the document in the Word**

- Click the File tab, and then click Options.
- Click Proofing.
- Under When correcting spelling and grammar in Word, make sure the Check grammar with spelling check box is selected.
- Select Show readability statistics.

After you enable this feature, open a file that you want to check, and check the spelling by pressing F7 or going to Review > Spelling & Grammar. When Word finishes checking the spelling and grammar, it displays information about the reading level of the document.

**Important:** You must correct, or Ignore, all spelling errors found in the document before the readability statistics will display. If there are still any red squiggles in the file, the readability statistics won't display.

**Recommendations**

- Translators should read the original versions very carefully to make sure if they fully understand the meanings. If the translator is not sure about the meaning of a work or sentence, they should contact the expert that research coordinator has introduced to her/him.
- After translation, please read the original version carefully and make sure that you don’t miss some information.
- Read out loud and edit the translated version several times, until you feel that it is readable and is easy to be understood by a native speaker.
